# No longer splitting hairs: an integrative mathematical model of tip growth

**DOI:** 10.1093/plcell/koag214

**Published:** 2026-07-18

**Authors:** Jan Wilhelm Schoeller

**Affiliations:** Assistant Features Editor, The Plant Cell, American Society of Plant Biologists; Unit of Plant Molecular Cell Biology, Institute for Biology I, RWTH Aachen University, Worringerweg 1, Aachen 52056, Germany

Plants drive their roots into the soil to harvest water and nutrients and transport these resources to the above-ground parts of plants. In this sense, the complete root system resembles a mine, with large main shafts and branching side tunnels. In this scenario, root hairs resemble narrow and short side workings that increase the root–soil interface. They emerge from root epidermal cells, and their growth is regulated by the targeted deposition of cell wall material at their tip. Similar to beams in a tunnel, the cell wall supports the root hair as it extends through the surrounding soil. The vacuole and cytoskeletal elements, including microtubules and actin filaments, provide additional structural support. Actin filaments also guide vesicles that deliver the components required for cell extension. In the mine analogy, actin filaments resemble rails along which vesicle “wagons” transport building materials to the advancing tunnel front.

The kinetics of root hair growth are conventionally divided into a fast-growing stage, with a mean tip growth rate of approximately 1.0 µm min^−1^ and a slow-growing stage, during which the tip growth rate declines to about 0.5 µm min^−1^ within 1 hour after the end of the fast-growing stage (Wang et al. 2020). The slow-growing stage continues for about 2 hours before growth arrest. During these stages, the distance between the nucleus and the root hair tip is tightly regulated by microtubules, while actin filaments promote slight oscillations of the nucleus that are required for proper tip growth.

Various computational models for polar tip growth exist. However, it has remained challenging to combine root hair elongation, cytoskeletal dynamics, and nuclear positioning in a single framework. In new work, Gilles Dupouy, Tamsin Spelman, and colleagues used real-time confocal imaging in microfluidic setups to measure tip growth rates and develop a mathematical model that incorporates cytoskeletal organization as well as nuclear movement and shape.

During microfluidic imaging of root hairs of the model plant species *Arabidopsis thaliana*, the authors observed an intermediate growth stage between the early fast-growing and late slow-growing stages. This transition stage emerged as a distinct third segment in a piecewise linear fit of tip growth kinetics measured in 20 root hairs from 12 individual plants. Imaging further revealed that cytoskeletal organization correlated with nuclear position. For example, during the transition stage, the nucleus moved closer to the root hair tip, while the peak of actin fluorescence intensity likewise shifted forward by about 20 µm (Fig. 1, “Experimental”). Microtubule fluorescence intensity, however, peaked behind the nucleus at the end of the fast-growing stage and then decreased considerably until growth arrest. Moreover, the tip-localized fringe of microtubules that has been observed in the fast-growing stage disorganizes during the transition stage, probably paving the way for the advancing nucleus.

**Figure 1:**
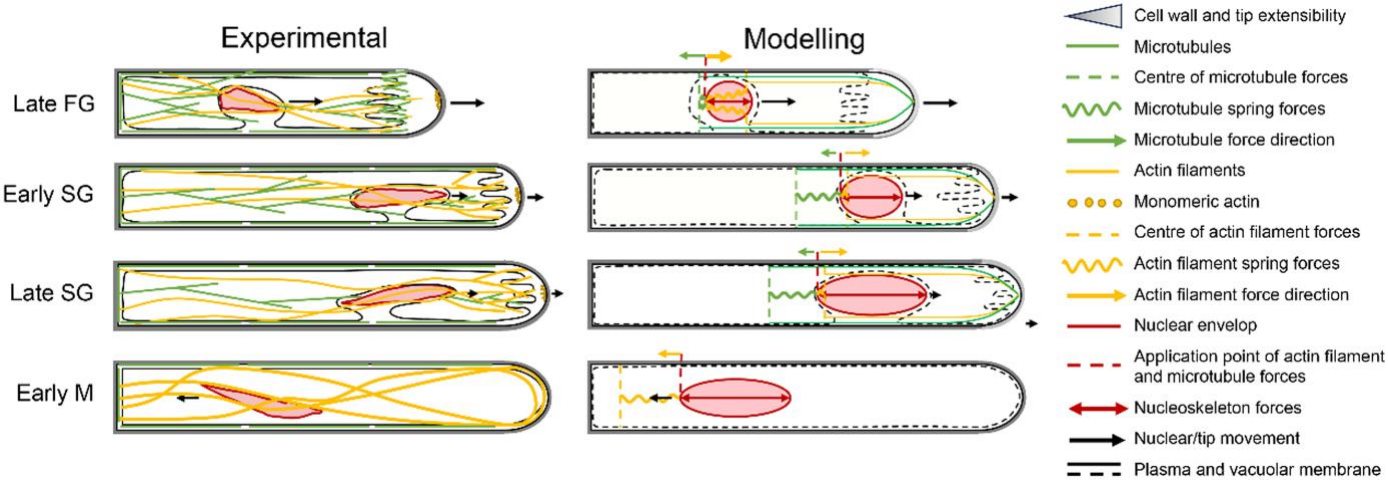
Phenotypic and mathematical model of root hair growth stages. Experimental and mathematical model as presented by Dupouy, Spelman, and co-workers (Dupouy et al. 2026). In the late fast-growing (FG) stage, the nucleus is pulled forward by actin filaments. During the transition phase in the early slow-growing (SG) stage, the nucleus moves further forward due to the declining contribution of microtubules and increased tension from actin filaments. In the late SG stage, the nuclear aspect ratio increases, driven by nucleoskeletal forces. In the early mature (M) stage, the nucleus reaches its maximum elongation. Following the disappearance of microtubules, the nucleus becomes fully disconnected from the tip, while backward forces from actin filaments cause nuclear retraction.

The authors translated their experimental data into a mathematical model, building on a published mechanical model for tip-growing cells (Dumais et al. 2006). In this new model, cell wall extensibility modulates tip growth rate, and nuclear dynamics are primarily controlled by forces exerted by microtubules and actin filaments. These cytoskeletal elements are represented as spring forces that either drive nuclear retraction away from the tip or promote nuclear progression toward the tip (Fig. 1, “Modeling”). With these components, the model accurately recapitulated experimentally observed root hair growth dynamics and nuclear movement.

They tested the predictive power of their model for microtubule dynamics and nuclear morphology. To this end, they compared simulations with growth data obtained from mutant plants and from plants subjected to pharmacological perturbations of cytoskeletal dynamics. The model captured many, but not all, of the experimentally observed effects. Altogether, the authors present a robust model for simulating root hair tip growth, while also showing that further integration of cytoskeletal complexity may improve model accuracy. They also emphasize the need to develop tools that can track turgor pressure as well as cell wall stiffness during root hair development. Within the mine analogy, this would translate to monitoring the reinforcements that prevent tunnel collapse, albeit at the molecular level. It will be interesting to see how the authors approach this challenge and whether they will implement recently developed tools, such as molecular rotor probes to visualize cell wall stiffness (Besten et al. 2025).

## Recent related articles in *The Plant Cell*

- He et al. (2025) propose that the *Magnaporthe oryzae* scaffolding protein *Mo*Spa2 promotes actin polymerization by organizing *Mo*Bni1-containing actin nucleation centers, thereby building polarized actin cables that support fungal tip growth and plant infection.
- Ke et al. (2025) reveal that the wheat inhibitor gene *B1*, together with *TaHDA6*, negatively regulates root hair length by modulating reactive oxygen species homeostasis, while loss of B1 function promotes longer root hairs and improved nutrient uptake.
- Li and Zhu (2024) show that polar localization and local translation of *ROP2* mRNA at the root hair tip establish proper ROP2 protein localization and thereby promote polarized root hair growth in *A. thaliana*.
- Yi et al. (2024) integrated photoconversion and binding kinetics into a mathematical model, explaining how phytochrome B–PIF complex formation can decrease at high red-light intensities and how this attenuation may help plants tune light and temperature responses.

## Data Availability

No new data were generated or analyzed in support of this article.
